# Curcumol Alleviates Obesity-Related Insulin Resistance and Inflammation in Skeletal Muscle via the SRC/PI3K/AKT Axis

**DOI:** 10.3390/nu18152431

**Published:** 2026-07-25

**Authors:** Yansong Fu, Xin Zeng, Bin Zhou, Jiayun Wang, Hong Qin

**Affiliations:** Department of Nutrition and Food Hygiene, Xiangya School of Public Health, Central South University, Changsha 410013, China; 226901016@csu.edu.cn (Y.F.); lpcp42@163.com (X.Z.); zb2323637560@163.com (B.Z.); zytdi00@163.com (J.W.)

**Keywords:** curcumol, SRC kinase, PI3K/AKT pathway, insulin resistance, inflammation, skeletal muscle, dietary bioactive compound, obesity

## Abstract

**Background/Objectives:** In obese skeletal muscle, impaired insulin signalling and persistent low-grade inflammation frequently arise together, jointly driving metabolic dysfunction; yet a single intracellular node capable of simultaneously correcting both defects has not been identified. Curcumol is the principal active sesquiterpene of the traditional Chinese herb *Curcuma zedoaria* (Christm.) Rosc. We therefore sought to determine whether curcumol modulates obesity-driven insulin resistance and inflammatory activation in skeletal muscle, and to delineate the responsible molecular pathway. **Methods:** The study combined in vivo and in vitro experiments with network pharmacology, molecular docking, pharmacological inhibition, and cellular thermal shift assay (CETSA). In vivo experiments used mice rendered obese by prolonged high-fat diet (HFD) feeding; in vitro, insulin resistance was modelled in C2C12 myotubes via palmitate challenge. Network pharmacology implicated SRC as a principal candidate target, and molecular docking assigned SRC kinase the highest binding affinity for curcumol. Selective blockade of SRC (PP2) and PI3K (LY294002) was used to delineate the signalling hierarchy. **Results:** Network pharmacology and molecular docking identified SRC kinase as the highest-ranked candidate target of curcumol. In both HFD-induced obese mice and palmitate-challenged C2C12 myotubes, curcumol restored SRC phosphorylation and activated the downstream PI3K/AKT axis, concurrently improving insulin sensitivity and attenuating NF-kB-driven inflammatory responses. Selective PI3K inhibition abolished all functional benefits of curcumol without altering SRC phosphorylation, whereas SRC blockade with PP2 prevented both PI3K/AKT activation and the downstream recovery of insulin sensitivity and inflammatory suppression, placing SRC upstream of PI3K/AKT in the signalling order. Direct binding of curcumol to SRC protein was confirmed by a cellular thermal shift assay. **Conclusions:** Curcumol directly engages SRC kinase and, through subsequent PI3K/AKT axis activation, concurrently rescues skeletal muscle insulin sensitivity and suppresses metabolic inflammation. These findings provide mechanistic justification for developing curcumol as a candidate dietary bioactive compound toward preventing and treating obesity-related metabolic disturbances.

## 1. Introduction

Global obesity prevalence has risen sharply over recent decades, with metabolic syndrome increasing proportionally; skeletal muscle dysfunction sits at the centre of this pathological trajectory [1,2,3]. Under euglycaemic-hyperinsulinaemic clamp conditions, skeletal muscle accounts for roughly 80% of whole-body insulin-stimulated glucose disposal, making it the primary insulin-responsive tissue and chief determinant of systemic glycaemic control [4,5]. In the setting of nutrient surplus and adiposity, skeletal muscle sustains two interrelated forms of injury [6]: reduced insulin-stimulated AKT phosphorylation disrupts GLUT4-vesicle mobilisation to the cell surface, thereby limiting glucose uptake [7,8,9]; and sustained activation of the NF-kB transcriptional programme promotes a state of chronic low-grade inflammation characterised by elevated production of TNF-alpha and IL-6 [10,11]. These processes mutually reinforce one another [12]: inflammatory mediators dampen upstream insulin receptor signalling, while the substrate burden imposed by insulin resistance further amplifies the local inflammatory milieu, compounding metabolic injury and predisposing individuals to secondary conditions such as type 2 diabetes mellitus and cardiovascular complications [13,14]. Existing pharmacological agents that target either insulin resistance or muscle-specific inflammation frequently exhibit inadequate tissue selectivity and dose-limiting systemic adverse effects [15].

PI3K/AKT signalling is the canonical intracellular relay through which insulin governs glucose homeostasis and, concurrently, is an important modulator of cellular inflammatory tone. Receptor engagement by insulin triggers membrane recruitment of PI3K, which catalyses the production of phosphatidylinositol-3,4,5-trisphosphate (PIP3); PIP3 in turn drives AKT phosphorylation, and activated AKT phosphorylates AS160 to relieve a constraint on GLUT4-vesicle exocytosis [15,16,17]. Attenuation of PI3K/AKT signalling is firmly established as a cardinal molecular feature of obesity-associated skeletal muscle insulin resistance. Simultaneously, PI3K/AKT contributes to inflammatory regulation under obese conditions [18], and mechanistic studies in macrophages and tumour cells have described distinct PI3K/AKT-driven pathways that limit pro-inflammatory cytokine production [19,20]; whether analogous mechanisms operate in obese skeletal muscle is less well defined. Given this dual involvement in insulin action and inflammatory modulation, the PI3K/AKT pathway represents a particularly compelling target for dietary bioactive compounds with broad pharmacological activity.

Growing preclinical evidence supports plant-derived compounds as effective modulators of both insulin signalling and metabolic inflammation in the context of obesity and type 2 diabetes [21]. Berberine activates AMPK in skeletal muscle of diet-induced obese animals, upregulates GLUT4 expression, and improves insulin-stimulated glucose disposal [22]. Resveratrol attenuates palmitate-induced skeletal muscle insulin resistance through AMPK/SIRT1-dependent mechanisms and reduces NF-kB-mediated inflammatory cytokine production [23]. Quercetin suppresses NF-kB-driven inflammatory signalling in obese skeletal muscle and restores GLUT4 expression in diet-induced obese rodents [24]. Curcumin modulates IRS-1/PI3K/AKT signalling to reduce peripheral insulin resistance in multiple animal models [21]. Collectively, these observations establish a precedent for exploiting phytochemical-kinase interactions to address the co-existing defects in insulin action and inflammatory tone that characterise obese skeletal muscle, and motivate the investigation of additional structurally distinct sesquiterpene candidates.

Curcumol, the principal sesquiterpene constituent of *Curcuma zedoaria* (Christm.) Rosc., possesses a broad pharmacological profile that includes anti-inflammatory and antioxidant properties [25,26]. Across diverse disease contexts—spanning obesity, hepatic steatosis, cardiac remodelling, and neuroinflammatory conditions—curcumol has repeatedly yielded beneficial metabolic and anti-inflammatory outcomes [27,28,29,30]. These converging observations raise the possibility that curcumol may benefit obese skeletal muscle; however, its direct molecular target and precise mechanistic pathway have not been established. SRC, the archetypal non-receptor protein-tyrosine kinase, serves as a versatile intracellular signalling hub through interactions with growth factor receptors and adaptor scaffolds; published evidence links it to both insulin receptor downstream signalling and inflammatory cascade regulation [31,32,33]. Drawing on these connections, the present work pursued three explicit aims: first, to determine whether curcumol alleviates obesity-induced skeletal muscle insulin resistance and low-grade inflammation in vivo and in vitro; second, to identify the primary intracellular target mediating these dual effects through integrated network pharmacology, molecular docking, and direct binding validation; and third, to delineate the downstream signalling sequence through which curcumol’s target engagement produces concurrent metabolic and anti-inflammatory benefits, thereby providing mechanistic justification for evaluating curcumol as a dietary bioactive candidate in obesity-related metabolic disease.

## 2. Materials and Methods

### 2.1. Reagents and Antibodies

Curcumol (CCM, purity ≥ 98%) was obtained from Hubei Yongkuo Technology Co., Ltd. (Tianmen, China). Palmitic acid (PA, purity ≥ 98%) was supplied by Sinopharm Chemical Reagent Co., Ltd. (Shanghai, China). Bovine serum albumin (BSA, purity ≥ 95%) was provided by MeilunBio (Dalian, China). The SRC inhibitor PP2, the PI3K inhibitor LY294002, and insulin were acquired from ABclonal Technology (Wuhan, China). Primary antibodies against *p*-PI3K, PI3K, *p*-NF-kB p65, NF-kB p65, *p*-AKT, AKT, TNF-alpha, IL-6, GLUT4, GAPDH, and beta-Tubulin were purchased from ABclonal Technology (Wuhan, China); primary antibodies against *p*-SRC and SRC were purchased from Wanleibio (Shenyang, China).

### 2.2. Animal Experiments

Male C57BL/6J mice (five weeks old; SPF grade; body weight 20 ± 2 g) were procured from Hunan SJA Laboratory Animal Co., Ltd. (Changsha, China). Animals were housed at 25 ± 2 degrees C with 50 ± 5% relative humidity under a 12 h light/dark photoperiod and allowed free access to food and water. Experimental diets (Research Diets, Inc., New Brunswick, NJ, USA) comprised a normal chow diet (NCD; D12450B; 10% of kilocalories from fat) and a high-fat diet (HFD; D12492; 60% of kilocalories from fat). Following one week of acclimatisation, mice were randomly assigned to either the NCD arm (*n* = 8) or the HFD arm (*n* = 16). From week 13, half the HFD animals were administered curcumol at 60 mg/kg/day by oral gavage (HFD + CCM group, *n* = 8), a dose in line with prior intervention studies [30,34,35]; the remaining HFD mice (*n* = 8) and all NCD animals received the same volume of 0.5% sodium carboxymethylcellulose vehicle. Treatment lasted four weeks. Prior to sacrifice, mice were fasted for 12 h; trunk blood was drawn for serum biochemistry, and gastrocnemius muscle tissues were rapidly excised for subsequent histological examination and molecular profiling. The gastrocnemius was selected because its predominantly fast-twitch fibre composition renders it particularly susceptible to changes in insulin signalling and inflammatory tone under obesogenic conditions. Metabolic assessments were performed at week 15. This 4-week duration was chosen for consistency with prior curcumol studies in metabolic disease models that documented robust phenotypic responses over comparable intervention periods [26,30]. Throughout the treatment phase, all HFD mice—both the vehicle-treated group and the curcumol-treated group—continued to receive the high-fat diet (D12492; 60% kcal from fat), ensuring that any beneficial effects of curcumol were evaluated against a background of persistent lipid overload and were not attributable to dietary change. For the GTT, food was withheld for 6 h before intraperitoneal glucose administration (2 g/kg body weight), and glycaemia was monitored at 0, 15, 30, 60, 90, and 120 min post-injection via tail-vein sampling. For ITT, mice fasted for 4 h received an intraperitoneal injection of insulin (0.75 U/kg body weight) and blood glucose was recorded at the same time points. To assess insulin-stimulated signalling, a subgroup received an intraperitoneal insulin challenge (1 U/kg body weight), and tissues were collected 15 min later; the remaining mice were not insulin-stimulated and served as the basal-state reference group. All procedures involving animals were carried out in strict accordance with ethical guidelines for laboratory animal use and received formal approval from the Institutional Animal Care and Ethics Committee of Xiangya School of Public Health, Central South University (Protocol No.: XYGW-2022-105; approval date: 27 December 2022).

### 2.3. ELISA

Blood samples were centrifuged at 1000× *g* for 10 min at 4 °C to collect serum. Serum TNF-alpha and skeletal muscle IL-6 concentrations were quantified using double-antibody sandwich ELISA kits (Mlbio, Shanghai, China). Briefly, standards or samples (50 µL per well) were transferred to the plate, followed by HRP-labelled detection antibody (100 µL per well); plates were subsequently incubated at 37 °C for 60 min. After the washing step, 50 µL each of chromogenic substrates A and B were pipetted in, and colour development proceeded at 37 °C for 15 min under light exclusion. The colorimetric reaction was terminated by adding 50 µL stop reagent per well. Optical density at 450 nm was recorded, and analyte concentrations were interpolated from the corresponding standard curves.

### 2.4. Cell Culture and Treatment

C2C12 murine skeletal myoblasts (Peking Union Medical College Cell Center, Beijing, China) were cultured in high-glucose DMEM supplemented with 10% fetal bovine serum at 37 °C in a humidified atmosphere containing 5% CO_2_. Upon reaching 80–90% confluency, myoblast differentiation was induced by replacing the medium with DMEM containing 2% horse serum for five days to generate mature myotubes.

Insulin resistance was established by challenging fully differentiated myotubes with palmitic acid (PA, 250 μM) for 24 h. The 250 μM concentration was selected on the basis of our previously published palmitate-challenge studies [36], in which an insulin-resistant phenotype characterised by impaired insulin-stimulated AKT phosphorylation and reduced glucose uptake was consistently reproduced, and further supported by the systematic review of Zimmerman et al. [37], which identified 250 μM as the most widely validated PA dose for this purpose across the C2C12 literature. This concentration also approximates the palmitate fraction of the elevated plasma free fatty acid pool documented in obese individuals [38], serving as a reliable and physiologically relevant challenge without inducing non-specific cytotoxicity. In the treatment protocol, cells first received PA (250 μM) or an equivalent volume of 1% BSA for 24 h, followed by curcumol (30 or 60 μM) for a further 24 h. For insulin-stimulated signalling experiments, cells were serum-deprived for 2 h before stimulation with 100 nM insulin for 30 min; cells not subjected to insulin treatment were retained to capture basal phosphorylation.

### 2.5. Oil Red O Staining

Intracellular lipid accumulation was assessed by Oil Red O staining. Fully differentiated C2C12 myotubes treated with PA (250 μM) or an equivalent volume of 1% BSA for 24 h were fixed in 4% paraformaldehyde for 10 min at room temperature, rinsed with 60% isopropanol, and then stained with 0.3% Oil Red O solution (prepared in 60% isopropanol) for 15 min at room temperature. After washing with 60% isopropanol to remove unbound dye, cells were counterstained with haematoxylin for 1 min. Representative photomicrographs were acquired under light microscopy (scale bar = 75 μm), and the stained lipid droplet area was quantified using ImageJ software (Version 1.8.0).

### 2.6. Cell Viability Assay

The cytotoxicity of curcumol (0–240 μM) was evaluated with and without co-exposure to PA (250 μM) by CCK-8 assay (AbMole, Shanghai, China). For each assay, cells were plated at 5 × 10^3^ per well into 96-well plates (60–70% initial density) and treated as outlined above. At the designated endpoint, CCK-8 reagent diluted 1:10 in DMEM (100 µL per well) was added to each well, and plates were returned to the incubator for 1 h at 37 °C. Absorbance at 450 nm was read on a multimode microplate photometer (Thermo Fisher Scientific, Waltham, MA, USA), and cell viability was expressed as a percentage of that in vehicle-treated control cells.

### 2.7. Glucose Uptake Assay

Cellular glucose uptake was assessed with a Glucose Uptake Assay Kit (Beyotime, Shanghai, China). After PBS washing, cells were pre-incubated in KRPH buffer for 40 min at 37 °C to deplete residual extracellular glucose. Insulin (100 nM) was then applied for 20 min, after which 2-deoxyglucose (2-DG; 10 mM) was added for an additional 20 min. After washing, cells were disrupted in Glucose Uptake Lysis Buffer and lysates cleared by centrifugation at 14,000× *g*, 4 °C, for 5 min. Equal volumes of clarified lysate and Glucose Uptake Detection reagent were combined and incubated for 30 min at 37 °C in the dark; the resulting absorbance at 450 nm was converted to intracellular 2-DG-6-phosphate (2-DG6P) content via a standard curve, serving as a surrogate index of glucose uptake.

### 2.8. Glycogen Synthesis Assay

Intracellular glycogen was measured with a Glycogen Assay Kit (Nanjing Jiancheng Bioengineering Institute, Nanjing, China). Cell pellets were rinsed with PBS, resuspended in phosphate buffer, and lysed by probe ultrasonication on ice. An aliquot was reserved for protein determination by BCA assay. A separate portion was mixed with alkaline reagent and heated to 100 °C for 20 min for polysaccharide hydrolysis; after cooling, double-distilled water was added to bring the volume to the required level. The resulting solution was reacted with anthrone reagent (sulfuric acid-based) and heated to boiling for 5 min before cooling to room temperature, and absorbance was recorded at 620 nm. Glycogen concentrations were normalised to total protein content.

### 2.9. Western Blot Analysis

Protein was extracted from frozen tissue pieces or harvested cell pellets using ice-cold RIPA lysis buffer (Beyotime, Shanghai, China) (30 min incubation); insoluble material was removed by centrifugation at 12,000 rpm for 15 min, and the clarified supernatants were retained for analysis. Protein concentrations were determined by BCA assay (GLPBio, Montclair, CA, USA), and 30 µg of protein per lane was resolved by SDS-PAGE before electrotransfer to PVDF membranes (Cytiva, Shanghai, China). After blocking with 5% skimmed-milk powder in TBST (Servicebio, Wuhan, China) for 1 h at room temperature, membranes were probed with the respective primary antibodies at 4 °C overnight. Following TBST washes, HRP-linked secondary antibodies were applied for 1 h at room temperature. Protein bands were visualised by enhanced chemiluminescence (GLPBio, Montclair, CA, USA) and captured digitally; densitometric quantification was performed using ImageJ software. For non-phosphorylated targets, band density was divided by that of the corresponding loading control (GAPDH or β-tubulin, as indicated per figure) on the same blot. For phosphorylated proteins, band density was first divided by the density of the corresponding total protein band, and the resulting ratio was then divided by the loading control density. All quantifications spanned *n* = 3–6 independent biological replicates. Under non-stimulated basal conditions, *p*-SRC, SRC, *p*-PI3K, PI3K, *p*-NF-kB p65, NF-kB p65, TNF-alpha, and IL-6 were assessed. After insulin stimulation, *p*-AKT and AKT expression levels were evaluated.

### 2.10. Immunofluorescence

Differentiated C2C12 myotubes plated in confocal dishes were treated as specified and subsequently stimulated with 100 nM insulin for 30 min. Cells were fixed using 4% paraformaldehyde solution for 15 min, after which non-specific binding was quenched with 5% BSA for 1 h at room temperature. The anti-GLUT4 primary antibody was incubated overnight at 4 °C; after washing, an Alexa Fluor 488-conjugated secondary antibody was applied for 1 h at room temperature. Nuclei were visualised by DAPI staining. Confocal fluorescence images were collected by laser scanning microscopy, with identical acquisition parameters maintained across all experimental groups. GLUT4 membrane signal intensities were quantified in ImageJ. For quantification, mean fluorescence intensity within the region of interest (ROI) was measured and normalised to total cell area (cell count per field) to correct for differences in cell density; background fluorescence was subtracted using a cell-free ROI.

### 2.11. Network Pharmacology Analysis

Candidate molecular targets of curcumol were retrieved from six public databases: SwissTargetPrediction, SuperPred, BATMAN, SEA, TCMSP, and ETCM. All outputs were unified through UniProt with removal of non-human entries, invalid records, and duplicates. Disease-related targets were independently retrieved from GeneCards and OMIM by searching the terms “obesity”, “skeletal muscle”, “insulin resistance”, and “inflammation”; data from both sources were pooled and deduplicated. Shared genes were identified using Venn diagram analysis, and a drug-target-disease interaction network was constructed in Cytoscape (Version 3.7.1). The overlapping gene set was uploaded to STRING for protein–protein interaction (PPI) network construction (species: Homo sapiens; confidence cutoff: 0.7); the exported network was visualised in Cytoscape and hub targets were prioritised by node degree. GO functional annotation and KEGG over-representation analyses of the shared gene list were performed in the R programming environment.

### 2.12. Molecular Docking

The X-ray crystallographic structure of SRC was obtained from the Protein Data Bank; co-crystallised ligands and water molecules were removed using PyMOL (Version 2.6.0). The 3D conformational structure of curcumol was retrieved from the PubChem compound database. AutoDock Vina (Version 1.5.6) was employed to perform molecular docking of curcumol against each hub target; calculated binding free energies were tabulated to rank binding preferences. The ligand structure was converted from SDF to PDB format using Open Babel and subsequently prepared in AutoDock Tools; target proteins derived from UniProt were processed by adding non-polar hydrogens, assigning Gasteiger partial charges, and defining appropriate grid box dimensions. Docking results were analysed, and protein-ligand interaction diagrams were produced in PyMOL and Discovery Studio (Version 2019).

### 2.13. Cellular Thermal Shift Assay (CETSA)

Fully differentiated C2C12 myotubes were treated with curcumol (60 μM) or vehicle (DMSO, equivalent volume) for 2 h. Cells were collected by scraping, washed, and resuspended in PBS, then equally distributed into PCR tubes; each tube was heated to a set temperature (37, 42, 47, 52, 57, 62, 67, 72, or 77 °C) for 5 min followed by rapid transfer to ice. Lysates were generated by three freeze–thaw cycles; insoluble debris was pelleted by centrifugation at 12,000 rpm for 20 min. Soluble SRC protein at each temperature point was detected by Western blot, and thermal denaturation curves were constructed by normalising each value to the 37 °C baseline signal (set as 100%) within each treatment condition.

### 2.14. Statistical Analysis

For in vivo studies, metabolic phenotyping measurements (GTT, ITT, body weight, and serum analytes) were obtained from *n* = 3–8 animals per group (the eight mice per group were shared across multiple experimental endpoints, with specific subsets allocated to individual assays); skeletal muscle Western blotting and histological analyses used *n* = 3–6 animals per group, with each blot performed in triplicate. Cell-based experiments comprised *n* = 3–6 independent biological replicates. All quantitative data are expressed as mean ± SEM. Immunofluorescence images were captured under identical laser intensity, gain, and exposure parameters across all groups. Immunoblot band densities were measured in ImageJ. *p* value below 0.05 was considered statistically significant.

## 3. Results

### 3.1. Curcumol Improves Insulin Resistance and Inflammation in HFD-Induced Obese Mice

**Figure 1:**
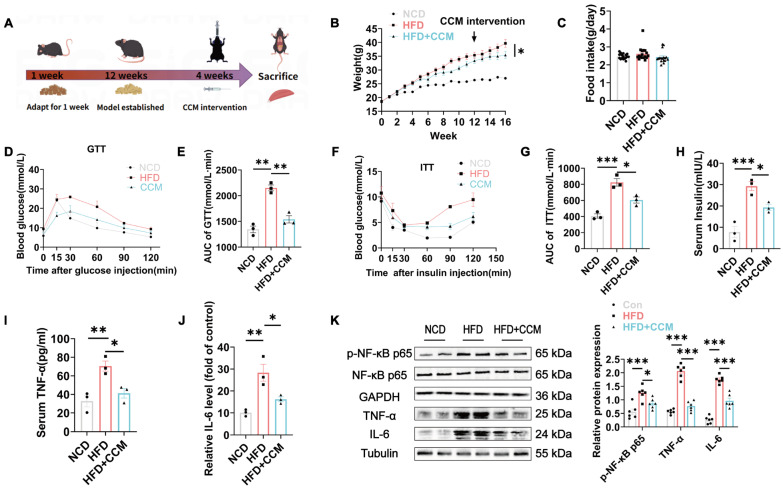
Curcumol improves insulin resistance and inflammation in HFD-induced obese mice. (**A**) Schematic diagram of the animal experimental protocol. (**B**) Longitudinal body weight curve (weeks 0–16). (**C**) Weekly food intake during the curcumol treatment phase. (**D**) Blood glucose curve from the intraperitoneal glucose tolerance test (GTT). (**E**) Area under the GTT curve. (**F**) Blood glucose curve from the insulin tolerance test (ITT). (**G**) Area under the ITT curve. (**H**) Serum insulin levels. (**I**) Serum TNF-alpha levels detected by ELISA. (**J**) Skeletal muscle IL-6 levels detected by ELISA. (**K**) Representative immunoblot images and semi-quantitative analysis of *p*-NF-kB p65, TNF-alpha, and IL-6 protein in skeletal muscle. *n* = 3–8 per group. Data are expressed as mean ± SEM. * *p* < 0.05; ** *p* < 0.01; *** *p* < 0.001 vs. HFD or Insulin group.

Body weight was monitored throughout the full experimental period (weeks 0–16). By week 12, HFD-fed mice had gained significantly more weight than NCD controls; curcumol treatment from week 12 onwards partially attenuated this progressive weight gain (Figure 1B). Weekly food intake during the 4-week treatment phase did not differ significantly between HFD and HFD + CCM groups (Figure 1C), indicating that the metabolic benefits of curcumol were not attributable to reduced energy intake.

GTT profiles revealed persistently elevated glycaemia in HFD mice versus NCD controls, with a correspondingly enlarged AUC; both parameters were substantially normalised by curcumol (Figure 1D,E). ITT data demonstrated pronounced whole-body insulin resistance in obese mice, a deficiency that curcumol substantially reversed (Figure 1F,G). Circulating fasting insulin concentrations were markedly higher in the HFD group than in NCD animals and decreased significantly following curcumol supplementation (Figure 1H). Serum TNF-alpha was likewise elevated in obese mice and was reduced by curcumol treatment (Figure 1I). Skeletal muscle IL-6 levels, determined by ELISA, were raised in HFD-fed animals and fell after curcumol administration (Figure 1J). Immunoblotting of gastrocnemius lysates showed markedly elevated *p*-NF-kB p65, TNF-alpha, and IL-6 in HFD animals; curcumol treatment reduced all three proteins (Figure 1K). Together, these results show that curcumol reverses HFD-induced insulin resistance and the accompanying inflammatory response in skeletal muscle in vivo.

### 3.2. Curcumol Improves Palmitate-Induced Insulin Resistance and Inflammation in C2C12 Myotubes

**Figure 2:**
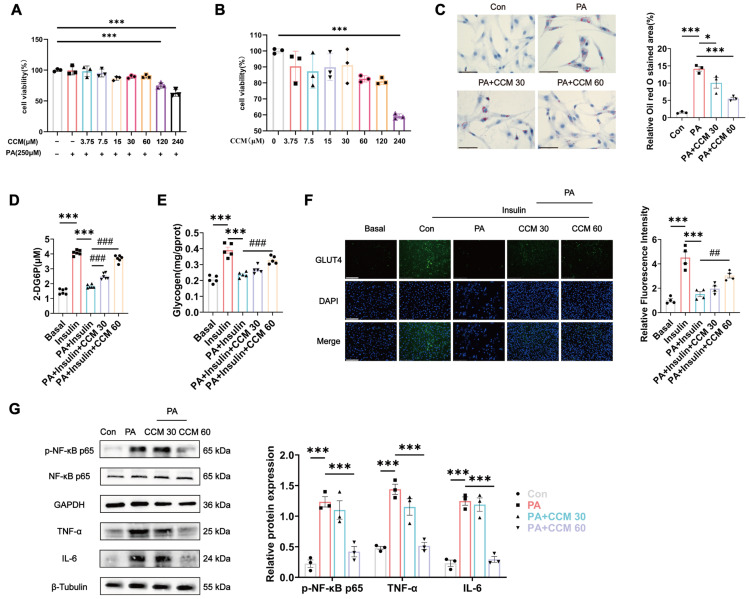
Curcumol improves palmitate-induced insulin resistance and inflammation in C2C12 myotubes. (**A**) Effect of curcumol on C2C12 cell viability in the presence of PA. (**B**) Effect of curcumol on C2C12 cell viability in the absence of PA. (**C**) Representative Oil Red O staining images and quantification of intracellular lipid accumulation in C2C12 myotubes (scale bar = 75 μm). (**D**) Glucose uptake in C2C12 myotubes. (**E**) Glycogen synthesis in C2C12 myotubes. (**F**) Representative immunofluorescence images and semi-quantitative analysis of GLUT4 membrane translocation in C2C12 myotubes (scale bar = 150 μm). (**G**) Representative immunoblot images and semi-quantitative analysis of *p*-NF-kB p65, TNF-alpha, and IL-6 protein in C2C12 myotubes. *n* = 3–6 per group. Data are expressed as mean ± SEM. * *p* < 0.05; *** *p* < 0.001 vs. Con, Basal, Insulin, or PA group; ## *p* < 0.01; ### *p* < 0.001 vs. PA + Insulin group.

To confirm the in vivo observations at the cellular level, a palmitate-induced insulin resistance model in C2C12 myotubes was employed. CCK-8 assays verified that curcumol was well tolerated at concentrations up to 60 μM irrespective of PA co-treatment (Figure 2A,B); 30 μM and 60 μM were consequently selected for subsequent mechanistic investigations.

Myotubes exposed to PA exhibited substantial deficits in insulin-stimulated glucose uptake and glycogen accumulation, impaired GLUT4 translocation to the plasma membrane, and concurrent rises in phospho-NF-kB p65 together with elevated TNF-alpha and IL-6 protein levels. PA-treated myotubes also showed markedly greater Oil Red O staining than BSA controls, validating effective lipid loading (Figure 2C). Curcumol countered all other palmitate-induced changes in a concentration-dependent manner, recovering glucose uptake and glycogen synthesis, promoting GLUT4 membrane localisation, and lowering inflammatory marker expression (Figure 2D–G). The close concordance between in vivo and in vitro observations substantiates the conclusion that curcumol mitigates lipid-overload-induced insulin resistance and inflammatory signalling in skeletal muscle.

### 3.3. Network Pharmacology and Molecular Docking Identify SRC as the Core Target of Curcumol for Improving Insulin Resistance and Inflammation

**Figure 3:**
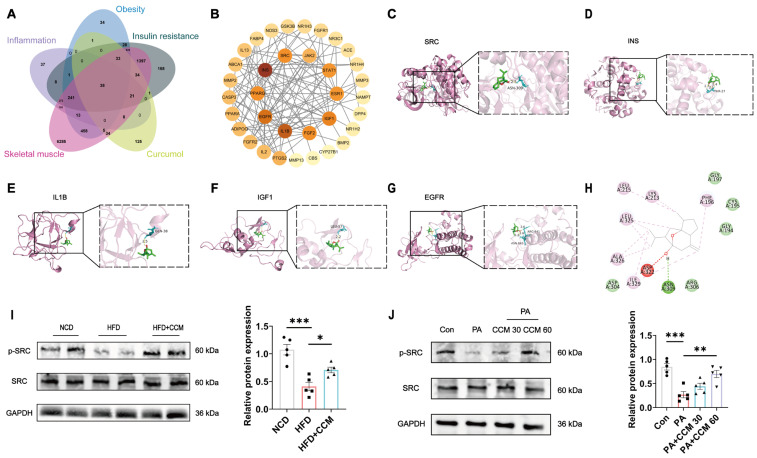
Network pharmacology and molecular docking identify SRC as the core target of curcumol for improving insulin resistance and inflammation. (**A**) Venn diagram of curcumol’s potential targets and disease targets for obesity, skeletal muscle, insulin resistance, and inflammation. (**B**) PPI network of overlapping targets; deeper colour indicates a higher degree value. (**C**–**G**) Lowest-binding-energy conformations of curcumol with each hub target. (**H**) Schematic of binding interaction forces between curcumol and SRC residues. (**I**) Representative immunoblot images and semi-quantitative analysis of *p*-SRC protein in mouse skeletal muscle. (**J**) Representative immunoblot images and semi-quantitative analysis of *p*-SRC protein in C2C12 myotubes. *n* = 5 per group. Data are expressed as mean ± SEM. * *p* < 0.05; ** *p* < 0.01; *** *p* < 0.001 vs. HFD or PA group.

To map the molecular basis of curcumol’s actions in obese skeletal muscle, we employed integrated network pharmacology and molecular docking. Cross-referencing curcumol candidate targets from six public databases against disease targets for insulin resistance, obesity, inflammation, and skeletal muscle yielded 38 shared genes (Figure 3A). Within the PPI network constructed from these overlap genes, SRC, INS, IL1B, IGF1, and EGFR occupied the highest-degree and highest-centrality positions, identifying them as the principal hubs of the curcumol-regulated network (Figure 3B). Molecular docking placed curcumol’s most favourable binding energy at SRC kinase relative to all other hub proteins (Figure 3C–G); the predicted binding pose was stabilised by hydrogen bonds and hydrophobic interactions with active-site residues (Figure 3H).

To functionally validate SRC as a relevant target, its phosphorylation status was examined under obese conditions. SRC phosphorylation was markedly diminished in the skeletal muscle of obese mice and in PA-treated myotubes alike—in keeping with impaired tyrosine kinase activity reported in needle biopsy samples from insulin-resistant human subjects—and curcumol restored phospho-SRC levels in both experimental contexts (Figure 3I,J). Taking together the evidence from network topology, docking energetics, and bidirectional functional pharmacology, SRC was designated as the primary molecular target responsible for curcumol’s dual protective effects.

### 3.4. The Protective Effects of Curcumol and SRC Are Associated with the PI3K/AKT Pathway

**Figure 4:**
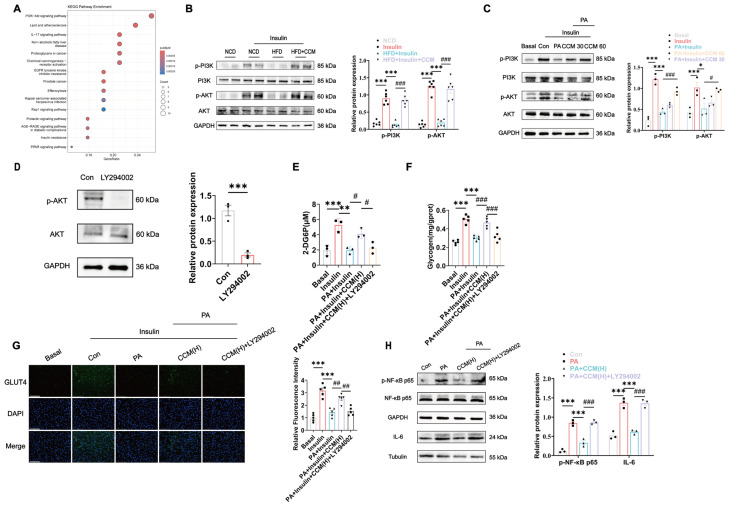
The protective effects of curcumol and SRC are associated with the PI3K/AKT pathway. (**A**) KEGG pathway enrichment analysis. (**B**) Representative immunoblot images and semi-quantitative analysis of *p*-PI3K and *p*-AKT protein in mouse skeletal muscle. (**C**) Representative immunoblot images and semi-quantitative analysis of *p*-PI3K and *p*-AKT protein in C2C12 myotubes. (**D**) Representative immunoblot images and semi-quantitative analysis of *p*-AKT protein in LY294002-treated C2C12 myotubes. (**E**) Glucose uptake in LY294002-treated C2C12 myotubes. (**F**) Glycogen synthesis in LY294002-treated C2C12 myotubes. (**G**) Representative immunofluorescence images and semi-quantitative analysis of GLUT4 membrane translocation in LY294002-treated myotubes (Scale bar = 150 μm). (**H**) Representative immunoblot images and semi-quantitative analysis of *p*-NF-kB p65 and IL-6 protein in LY294002-treated myotubes. CCM(H) denotes the high-dose curcumol group (60 μM). *n* = 3–6 per group. Data are expressed as mean ± SEM. * *p* < 0.05; ** *p* < 0.01; *** *p* < 0.001 vs. HFD, Basal, Insulin, or PA group; # *p* < 0.05; ## *p* < 0.01; ### *p* < 0.001 vs. HFD + Insulin, PA + Insulin, PA + Insulin + CCM(H) or PA + CCM group.

KEGG pathway enrichment analysis of the 38 shared targets positioned the PI3K/AKT signalling pathway at the head of the ranked list (Figure 4A), nominating it as the downstream effector arm through which curcumol’s activation of SRC translates into metabolic and anti-inflammatory benefits.

Consistent with these network predictions, phosphorylation of both PI3K and AKT was markedly suppressed in obese murine skeletal muscle and in PA-exposed myotubes; curcumol administration restored the phosphorylation of each kinase in both models (Figure 4B,C). To determine whether PI3K/AKT activity is indispensable for these protective effects, the selective PI3K antagonist LY294002 was co-administered with curcumol (Figure 4D). LY294002 completely abrogated the curcumol-conferred gains in glucose uptake (Figure 4E), glycogen synthesis (Figure 4F), and GLUT4 membrane localisation (Figure 4G), and similarly blocked the ability of curcumol to reduce phospho-NF-kB p65 and IL-6 protein (Figure 4H). Collectively, these results demonstrate that curcumol requires intact PI3K/AKT signalling to counteract insulin resistance and suppress inflammation through SRC in obese skeletal muscle.

### 3.5. SRC Is an Indispensable Target for the Dual Regulatory Effects of Curcumol

**Figure 5:**
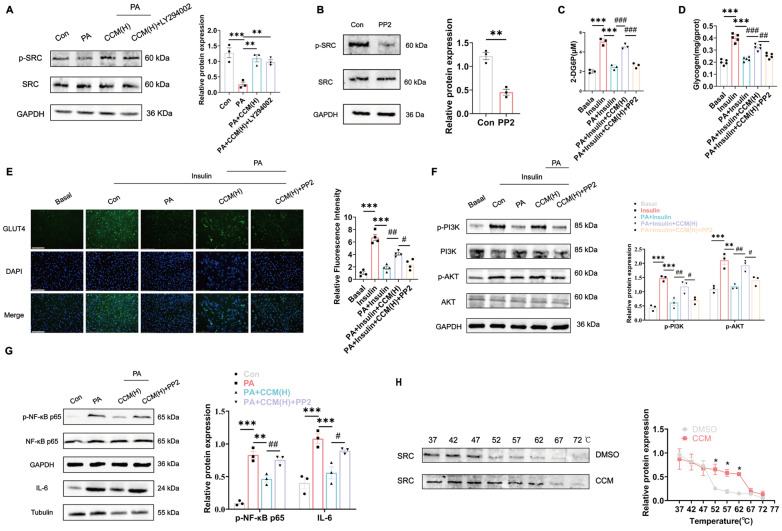
SRC is an indispensable target for the dual regulatory effects of curcumol. (**A**) Representative immunoblot images and semi-quantitative analysis of *p*-SRC protein in LY294002-treated C2C12 myotubes. (**B**) Representative immunoblot images and semi-quantitative analysis of *p*-SRC protein in PP2-treated C2C12 myotubes. (**C**) Glucose uptake in PP2-treated C2C12 myotubes. (**D**) Glycogen synthesis in PP2-treated C2C12 myotubes. (**E**) Representative immunofluorescence images and semi-quantitative analysis of GLUT4 membrane translocation in PP2-treated myotubes (Scale bar = 150 μm). (**F**) Representative immunoblot images and semi-quantitative analysis of *p*-PI3K and *p*-AKT protein in PP2-treated C2C12 myotubes. (**G**) Representative immunoblot images and semi-quantitative analysis of *p*-NF-kB p65 and IL-6 protein in PP2-treated C2C12 myotubes. (**H**) Representative CETSA immunoblot images and semi-quantitative analysis of curcumol binding to SRC. CCM(H) denotes the high-dose curcumol group (60 μM). *n* = 3–5 per group. Data are expressed as mean ± SEM. * *p* < 0.05; ** *p* < 0.01; *** *p* < 0.001 vs. Basal, Con, PA, Insulin, or DMSO group; # *p* < 0.05; ## *p* < 0.01; ### *p* < 0.001 vs. PA + Insulin + CCM(H) or PA + CCM group.

To define the epistatic relationship between SRC and PI3K/AKT, inhibitor experiments were performed. LY294002 abolished all curcumol-conferred functional benefits yet had no effect on SRC phosphorylation induced by curcumol (Figure 5A), demonstrating that SRC acts upstream and independently of PI3K. The SRC-selective inhibitor PP2 confirmed this signalling hierarchy: PP2 blocked curcumol-driven SRC phosphorylation (Figure 5B) and at the same time prevented curcumol from restoring PI3K and AKT phosphorylation (Figure 5F). PP2 also fully abolished the curcumol-mediated recovery of glucose uptake, glycogen synthesis, GLUT4 plasma membrane targeting, NF-kB p65 phosphorylation, and IL-6 expression (Figure 5C–G). These findings collectively establish SRC as an indispensable upstream node through which curcumol corrects skeletal muscle insulin sensitivity and mitigates inflammatory signalling.

Direct binding between curcumol and SRC was confirmed by CETSA. Relative to DMSO-treated controls, the SRC thermal denaturation curve was right-shifted in curcumol-exposed cells, indicating that ligand binding conferred measurable thermal stabilisation on the protein—a hallmark criterion of direct target engagement (Figure 5H). The data thus delineate a linear signalling hierarchy in obese skeletal muscle: curcumol binds and activates SRC, which then phosphorylates and activates the PI3K/AKT cascade, thereby normalising insulin responsiveness and restraining inflammatory gene expression.

## 4. Discussion

The simultaneous presence of insulin resistance and low-grade chronic inflammation in obese skeletal muscle is now broadly accepted as a principal driver of metabolic syndrome. Despite substantial advances in characterising the individual molecular events that underlie impaired insulin signalling and inflammatory activation, bioactive agents that can simultaneously normalise both defects through a single, pharmacologically tractable mechanism remain uncommon. By integrating network pharmacology with convergent in vivo and in vitro validation, we show here that curcumol directly binds SRC kinase and, through subsequent activation of the PI3K/AKT cascade, concurrently rescues insulin sensitivity and dampens inflammatory signalling in obese skeletal muscle. These findings add mechanistic depth and translational relevance to the growing body of evidence supporting dietary bioactive compounds as candidate metabolic therapeutics.

The reciprocal interplay between insulin signalling and the inflammatory machinery in skeletal muscle has been extensively documented [20]. Under obese conditions, intramyocellular accumulation of lipotoxic metabolites impairs proximal insulin signalling upstream of PI3K/AKT while simultaneously activating NF-kB-mediated transcription of pro-inflammatory cytokines such as TNF-alpha and IL-6 [39,40]. These secreted cytokines further attenuate PI3K/AKT activity through multiple serine kinase-based interference mechanisms, sustaining a mutually reinforcing cycle that progressively erodes metabolic homeostasis. This shared reliance on common signalling mediators makes these nodes logical pharmacological targets. Curcumol, the principal bioactive sesquiterpene of *Curcuma zedoaria* (Christm.) Rosc., has attracted growing interest as a multi-target natural product. Earlier studies demonstrated that curcumol reduces hepatic lipid burden in NAFLD models [30], curtails myocardial inflammatory injury post-infarction [29], and exerts anti-inflammatory and anti-fibrotic effects in hepatic stellate cells [35], collectively pointing to a broad pharmacological profile encompassing lipid metabolic regulation and inflammatory control. Building on that foundation, the present study confirms that curcumol corrects both pathological components in obese skeletal muscle: at the systemic level, it enhanced glucose tolerance, augmented insulin sensitivity, reduced fasting insulinaemia, and lowered skeletal muscle NF-kB p65 activity and IL-6 expression; at the cellular scale, it stimulated glucose uptake and glycogen deposition, promoted GLUT4 membrane translocation, and suppressed NF-kB p65 phosphorylation and IL-6 protein abundance. From a dietary perspective, these findings reinforce the view that plant-derived phytochemicals can target nutrient-sensing and inflammatory pathways, supporting the broader concept that food-based natural compounds represent accessible and generally well-tolerated strategies for managing obesity-linked metabolic dysfunction.

PI3K/AKT signalling is the canonical relay coupling insulin receptor engagement to glucose homeostasis: PI3K-derived PIP3 is required for AKT phosphorylation; activated AKT phosphorylates and inactivates GSK-3beta, thus facilitating glycogen synthesis [33]; and blunted AKT phosphorylation upon insulin stimulation is regarded as a defining hallmark of skeletal muscle insulin resistance [41]. Beyond metabolic control, AKT phosphorylation restrains TLR4-associated inflammatory signalling through FoxO1 transcriptional reprogramming, thereby curtailing pro-inflammatory cytokine production [42]; progressive PI3K/AKT attenuation in obese adipose tissue has additionally been associated with worsening local inflammation [43]. Taken together, this body of evidence positions the PI3K/AKT pathway as a shared regulatory hub governing both insulin sensitivity and inflammatory tone across metabolic tissues, yet direct mechanistic data from obese skeletal muscle specifically had been limited. The current study addresses this gap: network pharmacology identified PI3K/AKT as the topmost enriched pathway among curcumol-relevant targets; Western blotting documented substantially diminished PI3K and AKT phosphorylation in both obese mouse gastrocnemius and PA-treated C2C12 myotubes; and selective PI3K inhibition by LY294002 fully reversed all curcumol-driven improvements in insulin resistance and inflammation, confirming that PI3K/AKT activation is a mechanistically non-redundant step.

SRC, the archetypal member of the non-receptor tyrosine kinase superfamily, coordinates a wide array of intracellular signalling programmes [33], and accumulating evidence places it at the intersection of insulin receptor signal transmission and inflammatory cascade regulation [31,32], making it a logical node at which both pathological processes converge. In our network model, SRC consistently emerged as the hub with the highest connectivity among curcumol/disease target overlaps; computational docking yielded the most favourable binding energy score for the curcumol-SRC interaction relative to all other candidate hubs; and direct experimental assays in both the murine model and cultured myotubes revealed substantial SRC phosphorylation suppression under obese/lipotoxic conditions, in line with deficient tyrosine kinase phosphorylation reported in needle biopsy samples from insulin-resistant human subjects [44]. Pharmacological dissection clarified the epistatic order: LY294002 had no effect on curcumol-induced SRC phosphorylation, whereas PP2-dependent SRC blockade fully prevented curcumol from activating PI3K/AKT and eliminated every downstream benefit for insulin resistance and inflammation, unambiguously placing SRC upstream in the cascade. The metabolic consequences of SRC family kinase activity are clearly context-dependent: biochemical work has shown that SRC can amplify insulin receptor signalling through tyrosine phosphorylation of the receptor’s beta-subunit [32], whereas genetic ablation of the related family member Fyn was found to enhance fatty acid oxidation and preserve insulin sensitivity in diet-induced obese mice [45]. Such discrepancies most probably arise from the divergent substrate repertoires and tissue-restricted expression patterns of individual SRC family members [46]. Our data specifically establish that, within the pathological context of obesity-associated skeletal muscle, SRC kinase activity is indispensable for maintaining productive insulin signal propagation and containing pro-inflammatory responses.

A mechanistic caveat concerns our use of LY294002 to implicate PI3K in curcumol-induced AKT activation. LY294002 targets class I PI3K catalytic subunits but additionally inhibits mTOR and carries other off-target activities at micromolar concentrations routinely used in cell-based assays; its use therefore does not allow us to attribute the AKT response exclusively to PI3K. Two alternative or complementary routes to AKT activation warrant consideration. SRC has been shown to directly phosphorylate AKT at Tyr315 and Tyr326 in the kinase activation loop, with this tyrosine modification facilitating subsequent Thr308/Ser473 phosphorylation in a manner insensitive to LY294002 [47]. In addition, the scaffold protein beta-arrestin-2 couples SRC and AKT to the insulin receptor without requiring PI3K catalytic output: beta-arrestin-2 deletion selectively impairs insulin-stimulated AKT phosphorylation in liver and skeletal muscle while leaving PI3K activity intact, and adenoviral restoration of beta-arrestin-2 expression rescues insulin sensitivity in diabetic mice [48]; beta-arrestin-2 also negatively regulates NF-kB, consistent with the anti-inflammatory effects of curcumol observed here. Whether curcumol-activated SRC recruits beta-arrestin-2 as a co-scaffold to augment AKT activation beyond PI3K-dependent inputs, or whether direct SRC-to-AKT tyrosine phosphorylation contributes in parallel, cannot be resolved from the current data [47]. Future studies employing isoform-selective PI3K inhibitors, beta-arrestin-2 depletion models, and phosphospecific antibodies targeting AKT Tyr315/Tyr326 will be required to define the respective contributions of these routes.

Considered together, the present results demonstrate that curcumol simultaneously mitigates insulin resistance and inflammatory activation in obese skeletal muscle by engaging and activating the SRC/PI3K/AKT signalling axis, illustrating how a diet-derived natural compound can leverage endogenous kinase cascades to counter coincident metabolic defects. These data nominate SRC and the PI3K/AKT pathway as attractive targets for future pharmacological efforts focused on obesity-related skeletal muscle dysfunction. Several limitations warrant acknowledgement. PP2, though widely used as an SRC inhibitor, retains activity toward some off-target kinases; gene-silencing or conditional-knockout approaches targeting SRC specifically would enable more rigorous mechanistic validation. Additionally, inhibitor experiments were performed primarily at the cellular level; in vivo confirmation using pharmacological or genetic interventions will be important. Future work should examine whether comparable effects occur in primary human myotube preparations and whether curcumol acts synergistically with other bioactive dietary constituents in the context of metabolic disease. Direct measurements of glucose-stimulated insulin secretion were not obtained in this study; however, based on established in vivo phenotyping frameworks [49,50], the parallel ITT data implicate enhanced tissue insulin sensitivity as the principal driver of the improved glucose tolerance. Nonetheless, GSIS measurements in future work would fully delineate the contribution of β-cell function to curcumol’s metabolic effects. Energy expenditure was not directly measured; while food intake did not differ between HFD-vehicle and HFD + CCM groups during the treatment phase and animals were housed under uniform environmental conditions, curcumol may potentially improve metabolic homeostasis by elevating energy expenditure. Indirect calorimetry in future studies would provide a more complete account of curcumol’s influence on whole-body energy balance and expand the mechanistic understanding of its metabolic benefits beyond the SRC/PI3K/AKT axis.

## 5. Conclusions

Direct binding of curcumol to SRC kinase activates the downstream PI3K/AKT signalling axis, which simultaneously restores skeletal muscle insulin sensitivity and suppresses metabolic inflammation under obese conditions. These results provide mechanistic justification for a therapeutic strategy in which a dietary phytochemical corrects the dual pathology of obese skeletal muscle through engagement of a kinase signalling module, and lay the groundwork for further evaluation of curcumol as a candidate dietary intervention agent in obesity-associated metabolic disease.

## Data Availability

The public datasets used in this study are available from SwissTargetPrediction, SuperPred, BATMAN, SEA, TCMSP, ETCM, GeneCards, OMIM, PubChem, the Protein Data Bank, and STRING (see Section 2.11 and Section 2.12). The experimental data generated in this study are available from the corresponding author upon reasonable request.

## Abbreviations

The following abbreviations are used in this manuscript:

AKT	AKT serine/threonine kinase
CCM	Curcumol
CETSA	cellular thermal shift assay
GLUT4	glucose transporter type 4
GTT	glucose tolerance test
HFD	high-fat diet
IL-6	interleukin-6
ITT	insulin tolerance test
NCD	normal chow diet
NF-kB	nuclear factor kappa-light-chain-enhancer of activated B cells
PA	palmitic acid
PI3K	phosphoinositide 3-kinase
PPI	protein–protein interaction
SRC	proto-oncogene tyrosine-protein kinase Src
TNF-alpha	tumor necrosis factor-alpha
